# Quercetin‐Loaded Graphene Oxide Nanoparticles Modulate Inflammatory Gene Expression and Enhance Cell Migration In Vitro

**DOI:** 10.1002/open.202500215

**Published:** 2025-07-23

**Authors:** Hossain Alipour, Elnaz Tamjid, Mehrdad Behmanesh

**Affiliations:** ^1^ Department of Nanobiotechnology, Faculty of Biological Sciences Tarbiat Modares University P.O. Box: 14115‐154 Tehran Iran; ^2^ Department of Biomaterials, Faculty of Interdisciplinary Science and Technology Tarbiat Modares University Tehran Iran; ^3^ Department of Genetics, Faculty of Biological Sciences Tarbiat Modares University P.O. Box: 14115‐154 Tehran Iran

**Keywords:** graphene oxide, inflammation, quercetin, scratch assays, wound healing

## Abstract

Quercetin, a plant‐derived flavonoid, shows promising wound‐healing properties due to its antioxidant, anti‐inflammatory, and antibacterial effects. However, its limited water solubility limits its use. This study aims to enhance quercetin's efficacy by loading it onto graphene oxide (GO) and evaluating its in vitro effects for wound healing. GO is synthesized and quercetin is loaded onto its surface. Cytotoxicity of GO, quercetin, and quercetin‐loaded GO on human foreskin fibroblasts is determined. The expression levels of genes NF‐κB, IL‐1*β*, and TNF‐*α* are measured using qPCR. Wound healing is assessed via a scratch assay. The minimum inhibitory concentration (MIC) and maximum bactericidal concentration (MBC) of GO and quercetin‐loaded GO against *E. coli* and *S. aureus* are determined. Results show quercetin release is higher at pH 8.5 (59%) compared to pH 7.4 (40%). Cytotoxicity studies indicate that quercetin‐loaded GO enhances biocompatibility. The scratch assay shows a significantly higher wound closure rate in the quercetin‐loaded GO group after 48 h, than GO and quercetin alone (*p* < 0.05). Additionally, quercetin‐loaded GO exhibits antibacterial activity with MIC values of 4.8 μg mL^−1^ for both bacteria. These findings suggest that quercetin‐loaded GO is a promising candidate for wound healing.

## Graphical abstract

Herein, quercetin is loaded onto graphene oxide, and its biological properties are evaluated in vitro. The results show that the graphene oxide/quercetin nanocomposite reduces the expression of inflammation‐related genes and enhances fibroblast cell migration. Additionally, it exhibits antibacterial properties. Furthermore, the release of quercetin is pH‐sensitive. These factors can contribute to accelerating the wound healing process.

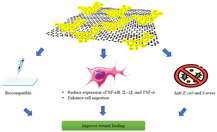

## 1 Introduction

According to reports, acute and chronic wounds are significant global health problems. Chronic wounds affect 1–2% of the world's population and impose a substantial economic burden on both individuals and healthcare systems.^[^ ^1^, ^2^ ^]^ The use of nanoparticles,^[^ ^3^ ^]^ and natural bioactive molecules,^[^ ^4^ ^]^ has emerged as a promising approach to accelerate wound healing and prevent infections in wound sites.

A skin wound is defined as a disruption of the skin's integrity, often caused by mechanical, chemical, thermal, or as a result of an underlying medical or physiological factor.^[^ ^5^, ^6^ ^]^ Wound healing is a well‐orchestrated physiological process, leading to the replacement of damaged tissue unless it is disrupted. Successful wound healing involves four distinct phases: hemostasis, inflammation, proliferation, and remodeling.^[^ ^7^ ^]^ Each phase can be impaired due to various factors such as nutritional deficiencies, underlying diseases, and infections, leading to chronic wounds.^[^ ^7^ ^]^ To accelerate the wound healing process, a variety of substances can be employed, for example, honey,^[^ ^8^ ^]^ silver nanoparticles,^[^ ^9^ ^]^ collagen,^[^ ^10^ ^]^ hyaluronic acid,^[^ ^11^ ^]^ plant crude extracts,^[^ ^12^ ^]^ and bioactive plant‐derived molecules.^[^ ^13^ ^]^ These compounds can be applied topically in various forms, such as sprays,^[^ ^14^ ^]^ creams,^[^ ^15^ ^]^ and dressings,^[^ ^16^ ^]^ to facilitate wound repair.

Quercetin, a flavonoid compound, has gained significant attention for its potential to enhance wound healing. Its antioxidant and anti‐inflammatory properties,^[^ ^17^, ^18^ ^]^ contribute to wound repair by promoting the polarization of macrophages from a proinflammatory to an anti‐inflammatory phenotype and modulating the immune response.^[^ ^19^ ^]^ Furthermore, quercetin exhibits antibacterial activity against a broad spectrum of bacteria, reducing the risk of wound infection.^[^ ^20^, ^21^ ^]^ However, its limited water solubility can hinder its efficacy.^[^ ^22^ ^]^ To overcome this limitation, the use of a suitable carrier to enhance its solubility and enable controlled release could offer substantial benefits in wound healing.

Graphene oxide, a 2D material composed of a honeycomb lattice of sp^2^‐hybridized carbon atoms, is a promising candidate for various biomedical applications, including drug delivery and tissue engineering. Its biocompatibility, antibacterial properties, and ability to interact with cellular components and facilitate tissue repair make it an attractive material for these applications. The polar nature of graphene oxide, attributed to the presence of hydroxyl, carboxyl, and epoxy functional groups makes it highly water soluble. Moreover, its exceptionally large surface area allows for the *π–π* stacking interactions with aromatic compounds such as quercetin, enabling its application as a carrier.^[^ ^23^, ^24^ ^]^ Moreover, graphene oxide can not only act as a carrier but also as an active agent contributing to wound healing.^[^ ^25^ ^]^

While other nanocarriers such as liposomes, polymeric nanoparticles, and chitosan‐based systems were indeed considered, each presented certain limitations in the context of our application. Liposomes, although widely used, can suffer from low physical stability, scalability issues, high production costs, and the risk of drug molecule outflow and fusion.^[^ ^26^, ^27^ ^]^ Polymeric nanoparticles may offer biocompatibility but often require more complex synthesis and offer less tunability in surface chemistry compared to graphene oxide (GO).^[^ ^28^, ^29^ ^]^ Chitosan‐based carriers, while advantageous in terms of biodegradability and mucoadhesion, are less suitable for efficient hydrophobic drug loading and may not form as stable drug‐carrier complexes.^[^ ^30^, ^31^ ^]^

This is the first in vitro study, to our knowledge, to examine the biological effects of quercetin‐loaded graphene oxide on wound healing. Following synthesis and characterization, graphene oxide was loaded with quercetin at an optimal concentration using absolute ethanol as a solvent. Subsequently, the release of quercetin at pH levels corresponding to normal and infected wounds was evaluated. The biocompatibility of the synthesized system was assessed by MTT assay on human foreskin fibroblasts (HFFs) as a normal fibroblastic cell. Cell migration as a crucial phase in wound healing, was examined via scratch assay. Given that inflammation is a vital stage in wound healing and chronic wounds often exhibit persistent inflammation, the effect of the developed nanosystem on the mRNA expression levels of IL‐1*β*, TNF‐*α*, and NF‐κB was evaluated to assess its potential for accelerating wound healing. Additionally, the minimum inhibitory concentration (MIC) and minimum bactericidal concentration (MBC) of the quercetin‐loaded graphene oxide were determined.

## 2 Experimental Section

The GO was synthesized based on the modified Hummer's method.^[^ ^32^ ^]^ Briefly, 0.5 g of graphite powder was dissolved in 50 mL of sulfuric acid (98%) and stirred for 40 min. Afterward, 0.5 g of NaNO_3_ and 3 g of KMnO_4_ were added and the mixture was stirred vigorously. Then, 100 mL of deionized water was added slowly into the solution at 35 °C while stirring for 30 min. As the solution color changed into dark brown, H_2_O_2_ was added and the reaction was terminated. After 24 h of precipitation, the supernatant was removed, and the precipitate was washed with 10% aqueous HCl, and then using deionized water several times. All chemicals were obtained from Sigma‐Aldrich (USA). The aqueous suspension was followed by bath sonication for 30 min, and finally, the GO powder was obtained by 36 h freeze‐drying. Next, the graphene oxide was characterized by UV–vis spectrophotometry (PG Instrument Ltd., Germany), FTIR (NICOLET FTIR 100 spectrophotometer over a range between 4000–500 cm^−1^), Raman (Teksan, Iran), and atomic force microscope (AFM; Autoprobe, CP‐Research, Veeco Instruments).

### 2.1 Quercetin Loading on Graphene Oxide and Characterizations

Prior to the loading experiment, different concentrations of quercetin were dissolved in absolute ethanol to ensure complete solubility and to determine the maximum soluble amount under the experimental conditions. To load quercetin onto the synthesized graphene oxide, three weight ratios of 1:1, 1:2.5, and 1:5 (Graphene oxide: Quercetin) were employed. Initially, graphene oxide was dispersed in absolute ethanol to prepare three separate suspensions at a concentration of 0.625 mg mL^−1^. The suspensions were magnetically stirred at room temperature for 1 h. In parallel, quercetin (Sigma‐Aldrich, USA) was dissolved in absolute ethanol by sonication (15 min) to enhance solubility and then added to each GO suspension to reach final quercetin concentrations of 0.625 mg mL^−1^, 1.562 mg mL^−1^, and 3.125 mg mL^−1^, respectively. After 10 min of sonication, the solutions were continuously stirred at 50 rpm for 24 h at room temperature in the dark to prevent quercetin degradation; to this end, the walls of the sample containers were carefully wrapped with aluminum foil to block exposure to ambient light. After incubation, the mixtures were centrifuged twice at 14,000 rpm for 20 min. The supernatants were carefully collected and filtered through 0.22 μm syringe filters to remove any suspended particles. Prior to UV–vis analysis, the collected supernatants were appropriately diluted. The absorbance of the diluted samples was then measured at 370 nm using a UV–vis spectrophotometer to determine the concentration of unbound quercetin. The percentage of quercetin loaded onto graphene oxide was then calculated using the following formula, where Wqi is the initial weight of quercetin used and Wqu is the weight of unbound quercetin in the supernatant.

(1) $$\text{Quercetin loading efficiency }(\%)\;=\;((W_{\text{qi}}-{\text{ W}}_{\text{qu}})/W_{\text{qi}})\;\times 100$$

To characterize the quercetin‐loaded graphene oxide, UV–vis, FTIR, and Raman spectroscopy were employed. Subsequently, the release of quercetin from graphene oxide was investigated at pH 7.4 and 8.5. After preparing the PBS solution (10 mM, pH 7.4) and adjusting the pH to ≈8.5 by adding NaOH, the quercetin‐loaded graphene oxide with known concentration was incubated at 37 °C under shaking. At predetermined time intervals, aliquots of the release medium were withdrawn, and an (equal) volume of fresh PBS was added. The withdrawn medium was then centrifuged at 14,000 rpm and the concentration of the released quercetin was quantified by measuring the absorbance at 370 nm using UV–vis spectroscopy and a standard curve (Figure S1, Supporting Information). Finally, the cumulative release of quercetin was calculated.

### 2.2 Cells Viability Assay

To evaluate the viability of HFF cells following treatment with different concentrations of graphene oxide, quercetin, and quercetin‐loaded graphene oxide, the MTT assay was employed. HFF cell line obtained from cell bank of the Pasteur Institute of Iran and cultured in high‐glucose Dulbecco's modified Eagle's medium (DMEM, Gibco) supplemented with 10% fetal bovine serum (FBS, Gibco), 100 unit mL^−1^ penicillin, and 100 μg mL^−1^ streptomycin in a cell culture flask and incubated at 37 °C with 90% of moisture and 5% of CO_2_. Cells were seeded at a density of 4 × 10^3^ cells per well of 96 well plates along with 100 ul of culture medium. After 24 h of incubation at 37 °C, the old medium was replaced with fresh medium containing various concentrations (1, 10, 25, 50, 100, and 150 μg mL^−1^) of graphene oxide, quercetin, and quercetin‐loaded graphene oxide. After incubation for 24, 48, and 72 h the treatment medium was discarded, cells were washed gently with PBS and 100 ul of MTT solution (5 mg ml^−1^ in PBS) was added to each well and the aluminum foil‐wrapped plate was then incubated for 4 h at 37 °C. Then to solubilize the formed formazan crystals, the MTT solution was removed and 100 ul of DMSO was added to each well and the plate was agitated using a shaker for 15 min. Finally, the absorbance of wells was measured at 570 nm using spectrophotometric plate Rader (Elisa Reader, BioTek, USA), as compared to the culture medium as a control.

### 2.3 Gene Expression Analysis

The effect of HFF cells treatment with 10 μg mL^−1^ of graphene oxide, quercetin, and quercetin‐loaded graphene oxide on the expression of NF‐κβ, IL‐1*β* and TNF‐*α* genes was investigated by qPCR. After 24 h of treatment, the total RNA of HFF cells was isolated with RiboEx (GenALL, Korea) according to the manufacturer's protocol. To eliminate DNA contamination from the total RNA, the extracted samples were treated with DNaseI (Sigma). The quality and quantity of the extracted RNA were assessed by 1% agarose (Sigma) electrophoresis gel and spectrophotometry, respectively. cDNA synthesis was performed using 3μg of total RNA with (Parstous, Iran) cDNA synthesis kit following the manufacturer's protocol. The expression of target genes was analyzed using the qPCR on the StepOne ABI system (Applied Biosystems, USA) with 5X Eva Green (Solis BioDyne, Estonia). GAPDH gene expression serves as an internal control. The primers used for gene expression analysis are listed in **Table** **1**. All reactions were performed at least in duplicate for each sample, and the specificity of PCR products was confirmed by performing a melt curve analysis and agarose gel electrophoresis. mRNA expression analysis of target genes was conducted using 2^−ΔCt^ and Fold Change methods with 2^−ΔΔCt^ calculation.^[^ ^33^ ^]^

**Table 1:** Primer sequences were used in this study to investigate gene expression changes using the qPCR technique.

Gene name	Accession No	Sequence of primer	Amplicon length
GAPDH	NM_002046.7	Forward: 5'‐AACGGGAAGCTCACTGGCATGG‐3'	306 bp
		Reverse: 5'‐CCACCACCCTGTTGCTGTAGCC‐3'	
TNF‐*α*	NM_000594.4	Forward: 5'‐GCAGTCAGATCATCTTCTCGAAC‐3'	182 bp
		Reverse: 5'‐GTAGATGAGGTACAGGCCCTCTG‐3'	
IL‐1*β*	NM_000576.3	Forward: 5'‐GATGTCTGGTCCATATGAACTG‐3'	294 bP
		Reverse: 5'‐CTGGGCAGACTCAAATTCCAGC‐3'	
NF‐κβ	NM_003998.4	Forward: 5'‐TACTCTGGCGCAGAAATTAGGTC‐3'	267 bp
		Reverse: 5'‐ACTGTCTCGGAGCTCGTCTATTTG‐3'	

### 2.4 Scratch Assay

To investigate in vitro wound healing ability of HFF cells under treatment with graphene oxide, quercetin, and quercetin‐loaded graphene oxide, the scratch assay was used. Herein, an appropriate number of cells were seeded on 12 well plates and incubated at 37 °C up to 70–80% of confluency. Then the old medium was replaced with the culture medium containing 1% of FBS and incubated for another 16 h for starvation. Next, a straight scratch was created in the center of each well using a sterilized pipette tip and the cells were gently washed twice with PBS. Then, 1 mL of culture medium (1% FBS) containing 10 μg mL^−1^ of each treatment was added to each well, and cells were incubated at 37 °C. Images were taken by a light microscope (Olympus IX53, Japan) after 24 and 48 h of incubation. The wound closure (%) was measured using ImageJ software (Version 1.54 g, National Institutes of Health, USA) and Equation (2). In this equation W_0_ and W_t_ represent the gap size immediately after and predetermined time, respectively.

(2) $$\text{Wound closure }\left(\%\right)\;=\;\left(\left(W_{0}-W_{t}\right){\text{/W}}_{0}\right)\;\times 100$$

### 2.5 Determination of MIC and MBC

To determine the MIC and MBC of graphene oxide and quercetin‐loaded graphene oxide against *E. coli* (ATCC 25922) and *S. aureus* (ATCC 25923), a broth microdilution assay was performed. Therein, the bacterial suspensions were prepared at a 0.5 McFarland standard. Serial twofold dilutions of the treatments in concentrations ranging from 2.5 mg mL^−1^ to 2.4 μg mL^−1^ were prepared in Mueller–Hinton broth and inoculated with the bacterial suspensions in 96 well plates. The control well contained Mueller–Hinton broth along with the bacterial suspension. After 24 h of incubation at 37 °C, the MIC was determined as the lowest concentration at which no visible growth was observed. To determine the MBC, a 50 ul aliquot of all wells that showed no visible bacterial growth was seeded on agar plates and incubated for 24 h at 37 °C. MBC was considered the lowest concentration killing 99.9% of the bacterial population.

### 2.6 Statistical Analysis

Data were analyzed using GraphPad Prism version 8.4.3(GraphPad, San Diego, CA) which was expressed as the mean ± standard deviation. Two‐way ANOVA and Tukey's multiple comparison test were applied for the measurement of statistical difference, *p* value < 0.05 indicates a statistically significant result.

## 3 Results and Discussion

### 3.1 Synthesis of Graphene Oxide and Quercetin Loading

**Figure 1:**
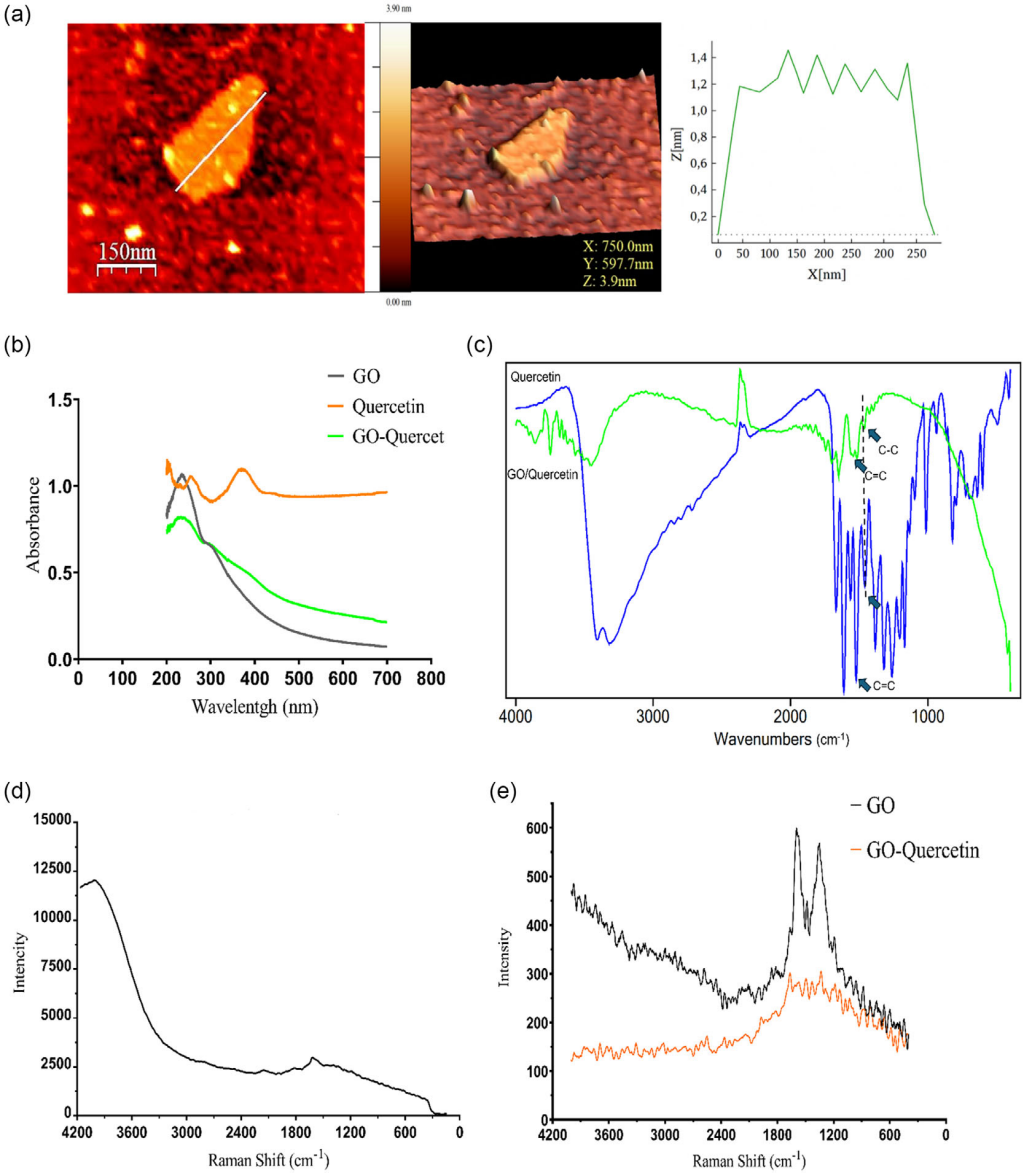
a) AFM image and dimensions of GO. b) UV–vis spectra of GO, quercetin, and quercetin‐loaded GO. c) FTIR of quercetin and quercetin‐loaded GO. d) Raman spectra of quercetin. e) Raman spectra of GO and quercetin‐loaded GO.

In this study, graphene oxide was initially synthesized using a modified Hummers method. The AFM image of the synthesized graphene oxide, presented in **Figure** **1**a, reveals an individual graphene oxide sheet with a thickness of less than 2 nm. Considering that the thickness of a single‐layer graphene oxide is reported to be around 0.4–1.7 nm,^[^ ^34^ ^]^ the synthesized graphene oxide in this research is considered a single layer. Figure 1b shows the UV–vis spectra of quercetin, graphene oxide, and quercetin‐loaded graphene oxide. In the graphene oxide spectrum, the absorption at around 230 nm corresponds to the C=C bond (π–π* electronic transition) and the absorption at around 300 nm corresponds to the C=O bond (n–π* electronic transition), confirming the synthesis of graphene oxide.^[^ ^35^ ^]^ The loading of quercetin onto graphene oxide nanosheets causes a decrease in the peak intensity in the 230 nm region. Additionally, the appearance of the characteristic quercetin peak in the 370 nm region of the graphene oxide spectrum indicates the attachment of quercetin to graphene oxide. To characterize the functional groups and the interaction of quercetin with graphene oxide, FTIR spectroscopy was used. The FTIR spectrum of graphene oxide is shown in Figure S2 and the spectrum of quercetin and quercetin‐loaded graphene oxide is shown in Figure 1c. In the spectrum of graphene oxide‐loaded quercetin, the broad peak around 3450 cm^−1^ corresponds to the O—H bond present in water and results from the oxidation of nanosheets. The peak observed around 1720 cm^−1^ corresponds to the C=O stretching vibration in the carboxyl group, and the peak observed around 1630 cm^−1^ corresponds to the asymmetric stretching vibration of the C=C bond in the unoxidized regions of graphite.^[^ ^36^ ^]^

The peak observed around 1520 cm^−1^ corresponds to the stretching vibration of the C=C bond in the A ring of quercetin, which appeared in the graphene oxide spectrum.^[^ ^37^ ^]^ Also, the peak observed around 1460 cm^−1^ corresponds to the stretching vibration of the C—C bond, which appeared in the graphene oxide spectrum,^[^ ^38^ ^]^ which could be an indication of the *π–π* interaction between quercetin and graphene oxide. According to the results of Raman spectroscopy, shown in Figure 1d,e, the main Raman peak of quercetin is observed at 1615 cm^−1^, which corresponds to the stretching vibration of the aromatic ring of this molecule. This peak is observed at 1616 cm^−1^ after loading quercetin onto graphene oxide nanosheets, and it has undergone a slight blue shift. Similarly, the intensity of the weak peaks corresponding to in‐plane vibrations observed in the 800–850 and 900–1100 cm^−1^ regions has increased. These findings confirm the attachment of quercetin to graphene oxide through *π–π* interactions.^[^ ^37^ ^]^

Quercetin, a natural bioactive molecule, exhibits diverse biological properties including antioxidant, anti‐inflammatory, anticancer, and antimicrobial activities. However, its limited solubility in water and physiological environments has restricted its application.^[^ ^39^ ^]^ Nevertheless, loading it onto nanocarriers can help overcome this issue, enhancing its bioavailability and prolonging its effects through sustained release.^[^ ^40^ ^]^ In this study, three weight ratios of 1:1, 1:2.5, and 1:5 (graphene oxide: quercetin) were used to load quercetin onto graphene oxide. The results showed that the highest loading of quercetin onto graphene oxide was observed at a weight ratio of 1:1, reaching 89.99% ± 0.47. The loading of quercetin onto graphene oxide at ratios of 1:2.5 and 1:5 was 87.74% ± 0.94 and 89.57% ± 0.7, respectively (**Table** **2**). Based on these results, a ratio of 1:1 was used for subsequent experiments to load quercetin onto graphene oxide.

**Table 2:** Loading efficiency (%) of quercetin on GO.

GO: Quercetin [W: W]	Loading efficiency [%]
1:1	89.99 ± 0.47
1:2.5	87.74 ± 0.95
1:5	89.57 ± 0.7

**Figure 2:**
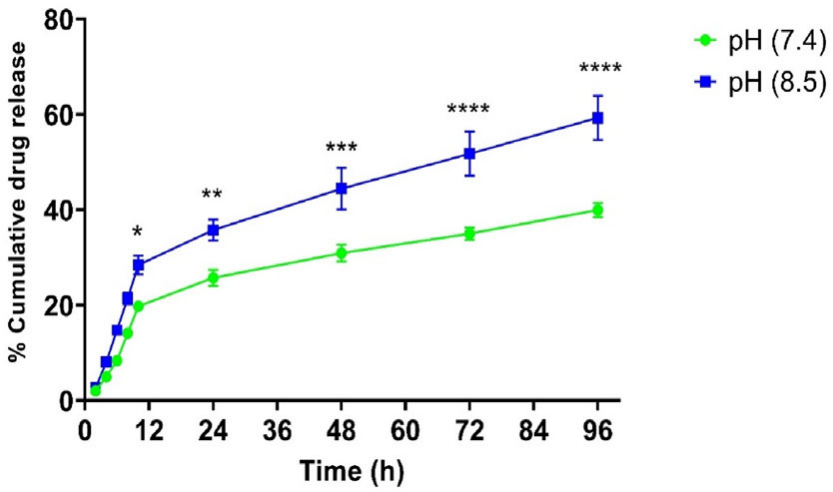
Cumulative quercetin release (%) from GO at pH 7.4 and 8.5.

Subsequently, the release of quercetin from graphene oxide was investigated at pH 7.4 and 8.5. According to the drug release profile in **Figure** **2**, in both groups, an initial burst release of quercetin from graphene oxide was observed within the first 12 h which could be attributed to the release of molecules with weaker bonds to graphene oxide. Additionally, the amount of quercetin released from graphene oxide at pH 8.5 was significantly higher than at pH 7.4 (28% vs. 19%). However, a sustained release was observed at further times. Additionally, the results showed that the amount of quercetin released from graphene oxide at pH 8.5 was significantly higher in comparison to pH 7.4 at subsequent time points. After 96 h, ≈59% of quercetin was released at pH 8.5 and about 40% at pH 7.4. The higher release of quercetin from graphene oxide at alkaline pH could be due to the increased negative charge and weakening of *π–π* interactions, as well as the increased solubility of quercetin at higher pH values.^[^ ^41^, ^42^ ^]^ Previous studies have shown that the pH in chronic wounds increases and may even reach 9, which could indicate a bacterial infection.^[^ ^43^, ^44^, ^45^, ^46^ ^]^ Notably, the pH‐sensitive release of quercetin from graphene oxide could be beneficial in treating chronic and infected wounds as it would (lead) to increased release of quercetin and thus enhance its anti‐inflammatory, antioxidant, and antibacterial effects, which are of great importance in improving all wound healing phases.^[^ ^47^ ^]^

### 3.2 Cells Viability

**Figure 3:**
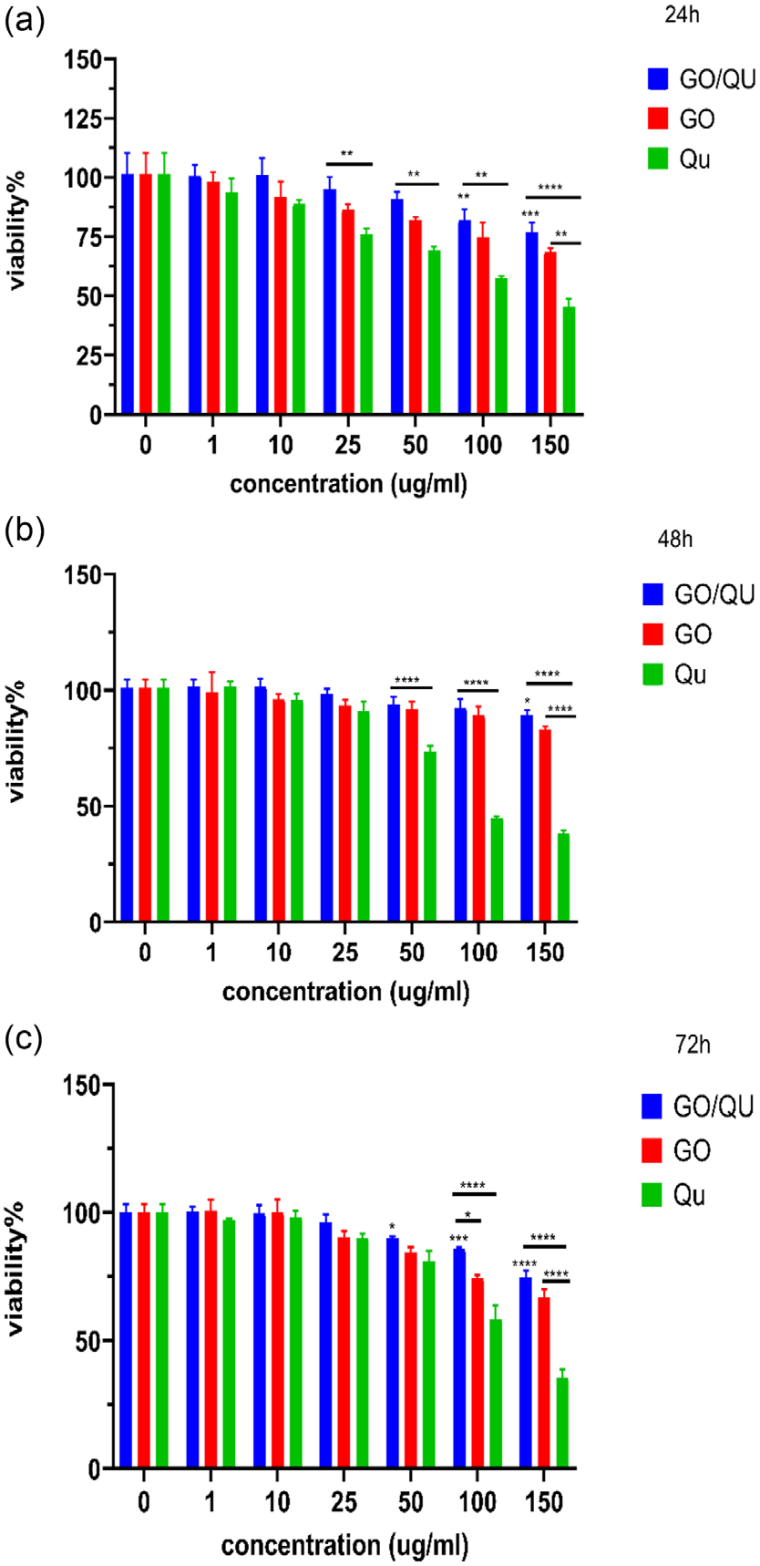
Fibroblasts viability under treatment with different concentrations of GO, quercetin, and quercetin‐loaded GO after a) 24, b) 48, and c) 72 h.

In this study, the MTT assay, a colorimetric method for assessing cell viability,^[^ ^48^ ^]^ was employed to investigate the effects of treating HFF cells with concentrations ranging from 1–150 μg mL^−1^ of graphene oxide, quercetin, and quercetin‐loaded graphene oxide after 24, 48, and 72 h of treatment. The results are shown in **Figure** **3**. Based on the obtained results, cell viability at each time point decreased in a dose‐dependent manner with increasing concentrations of the studied compounds. It is worth noting that, cell viability after 24 h of treatment with quercetin‐loaded graphene oxide, significantly decreased at concentrations of 100 (*p* value = 0.0054) and 150 (*p* value = 0.0001) μg mL^−1^, with values of 83.11% ± 3.7 and 78.41% ± 3.3, respectively. However, after 48 h of treatment, cell viability decreased slightly only at a concentration of 150 μg mL^−1^ (*p* value = 0.0171). Finally, after 72 h of treatment, cell viability decreased significantly at concentrations of 50 (*p* value = 0.0319), 100 (*p* value = 0.0004), and 150 (*p* value < 0.0001) μg mL^−1^. Additionally, the results indicate that cell viability increased when quercetin was loaded onto graphene oxide compared to graphene oxide alone at the same concentration. According to previous studies, quercetin is a potent antioxidant with anti‐inflammatory and anticancer properties that exert its effects by influencing enzymes, intracellular signaling pathways, and reactive oxygen species.^[^ ^17^, ^49^, ^50^, ^51^ ^]^ On the other hand, it has been shown that the toxicity of graphene oxide can be due to the generation of oxidative stress.^[^ ^52^, ^53^ ^]^ Therefore, quercetin can (lead) to increased viability compared to graphene oxide alone at the same concentrations, by reducing oxidative stress. Finally, considering that cell viability at a concentration of 10 μg mL^−1^ of quercetin‐loaded graphene oxide is almost identical to the control group, this concentration was selected for further studies.

### 3.3 Gene Expression Analysis

**Figure 4:**
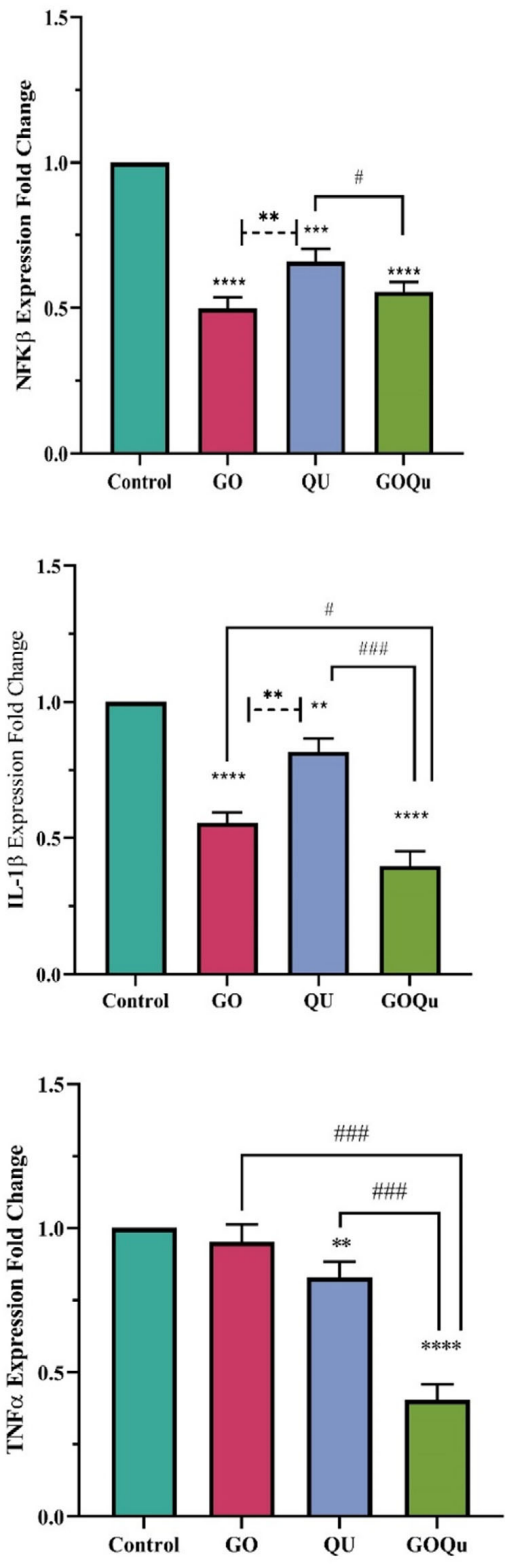
Expression of NF‐κB, IL‐1*β*, and TNF‐*α* after treatment with 10 μg/mL of GO, quercetin, and quercetin‐loaded GO.

Inflammation is a crucial phase of wound healing. However, chronic inflammation can arise if this process is disrupted. Inflammatory cytokines play a pivotal role in regulating inflammation at the wound site. One objective of this study was to examine the effect of quercetin‐loaded graphene oxide on the gene expression of the inflammatory cytokines IL‐1*β* and TNF‐*α* in fibroblast cells. Additionally, NF‐κB, a transcription factor with a crucial role in regulating inflammatory signaling pathways, was selected as a candidate gene in this study. Impaired fibroblast function can hinder wound healing, as fibroblasts contribute to chronic inflammation by producing and secreting inflammatory cytokines.^[^ ^54^ ^]^ Therefore, compounds that can reduce intracellular inflammation and cytokine secretion from fibroblasts may promote wound healing. As shown in **Figure** **4**, treatment of HFF cells with graphene oxide significantly reduced the expression of NF‐κB and IL‐1*β*, nonetheless did not affect TNF‐*α* expression. These results are consistence with those of Hoyle et al., who investigated the effect of graphene oxide on immortalized bone‐marrow‐derived macrophages. However, in our study, fibroblasts were examined. Similar to our findings, Hoyle et al. observed a significant decrease in IL‐1*β* release at a concentration of 10 μg mL^−1^ of graphene oxide, while TNF‐*α* levels remained unaffected. However, in their study, the NF‐κB pathway was not specifically targeted, and the decrease in IL‐1*β* was attributed to metabolic changes in macrophages.^[^ ^55^ ^]^ In contrast, our study demonstrates that graphene oxide significantly reduces NF‐κB gene expression in fibroblasts, which could (lead) to a decrease in the expression of inflammatory cytokines like IL‐1*β*.^[^ ^56^ ^]^ Previous studies have shown that the effects of graphene oxide can vary depending on the cell type and physicochemical properties of the material.^[^ ^57^ ^]^ Our results indicate that quercetin also significantly reduced the expression of all three genes examined in this study. The most significant reduction was observed in the expression of NF‐κB, which may contribute to the decreased expression of TNF‐*α* and IL‐1*β*. As these two inflammatory cytokines play a crucial role in chronic wound formation, their reduced expression can positively impact wound healing. Previous studies have shown that quercetin inhibits the inflammatory effects of TNF‐*α* on HUVEC, IEC, thymocyte, and splenocyte cell lines by inhibiting the NF‐κB pathway.^[^ ^58^, ^59^, ^60^ ^]^ Our results demonstrate that quercetin also reduces NF‐κB gene expression in fibroblasts, which could contribute to reducing inflammation in chronic wounds. Loading quercetin onto graphene oxide led to a significant decrease in the expression of all three genes examined. Quercetin‐loaded graphene oxide had a greater inhibitory effect on NF‐κB expression compared to quercetin alone, although no significant difference was observed compared to graphene oxide alone. Additionally, the expression of TNF‐*α* and IL‐1*β* was significantly lower in cells treated with quercetin‐loaded graphene oxide compared to cells treated with quercetin or graphene oxide alone, indicating a synergistic effect. As shown in the figure for TNF‐*α*, graphene oxide alone did not reduce the expression of this gene, but loading it with quercetin significantly reduced its expression. Furthermore, a synergistic effect of quercetin‐loaded graphene oxide was observed in reducing IL‐1*β* expression. This enhanced reduction in gene expression in quercetin‐loaded graphene oxide can be attributed to the combined anti‐inflammatory effects of both molecules and the potential of graphene oxide to improve the solubility, stability, and sustained release of quercetin. These factors may contribute to the prolonged effects of quercetin.

### 3.4 In Vitro Wound Healing

**Figure 5:**
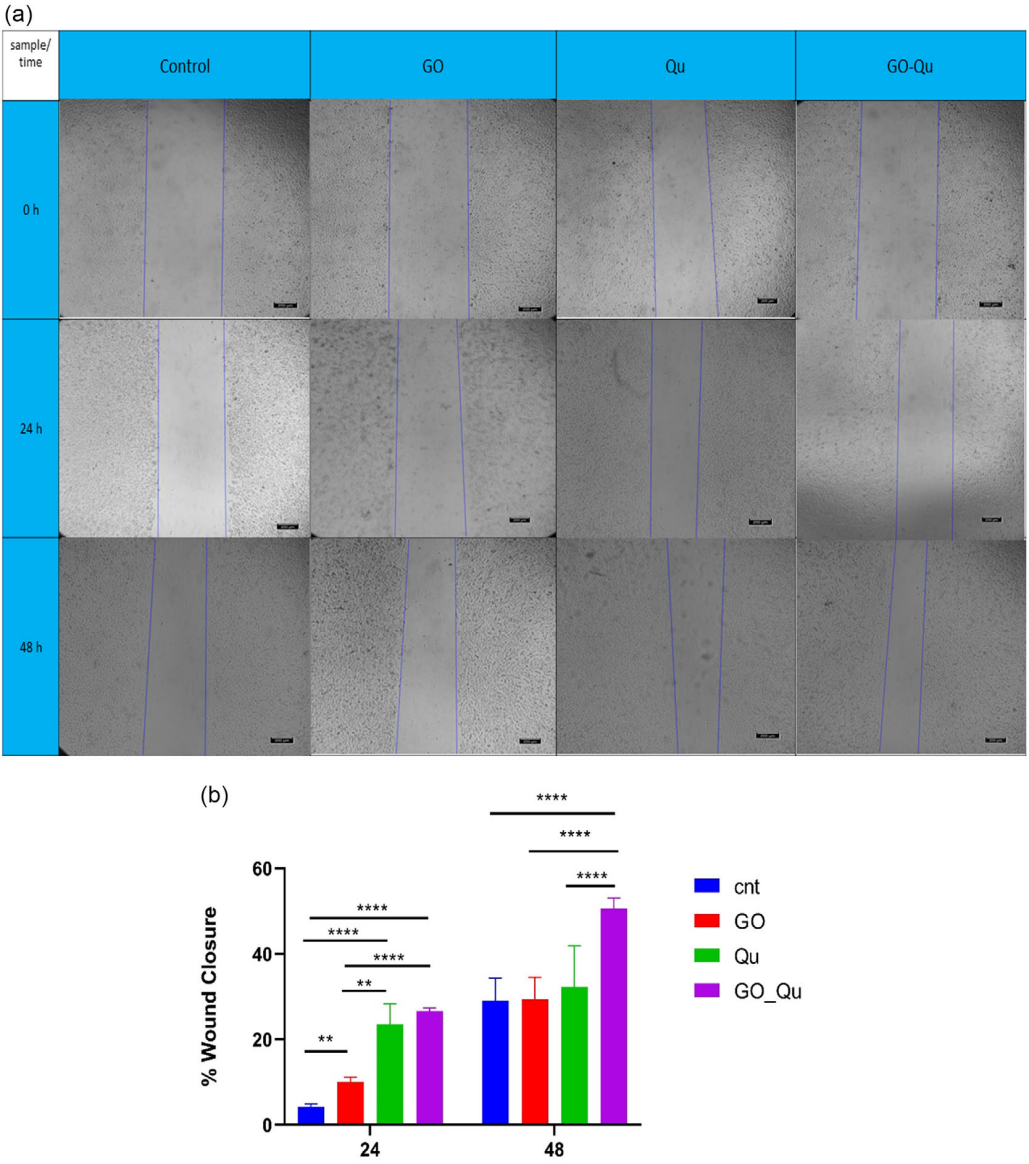
a) Photographs of fibroblast migration and b) wound closure (%) after 24 and 48 h of treatment with GO, quercetin, and quercetin‐loaded GO.

Fibroblasts play a crucial role in wound healing. They migrate to the wound site, synthesize extracellular matrix, secrete growth factors, and contribute to granulation tissue formation, all of which are essential for tissue repair. Impaired fibroblast migration to the wound area can disrupt wound healing and (lead) to chronic wounds.^[^ ^61^ ^]^ Therefore, compounds that can accelerate fibroblast migration to the wound site may have a positive impact on wound healing. In this study, the effect of graphene oxide, quercetin, and quercetin‐loaded graphene oxide on the migration of HFF cells after 24 and 48 h of treatment was investigated. As shown in **Figure** **5**a,b, after 24 h of treatment, graphene oxide, quercetin, and quercetin‐loaded graphene oxide significantly increased cell migration compared to the control group. They closed ≈10 ± 0.96%, 24.8 ± 4.30%, and 26.6 ± 0.66% of the initial wound, respectively. Quercetin significantly enhanced cell migration compared to graphene oxide (*p* < 0.05). However, no significant difference was observed in cell migration between quercetin and quercetin‐loaded graphene oxide. After 48 h of treatment, no significant difference was observed between the graphene oxide and quercetin‐treated groups compared to the control group. However, treatment with quercetin‐loaded graphene oxide significantly increased cell migration (*p* < 0.05) and closed (49.3 ± 2.18%) the initial wound as compared to all other groups. Regarding the effects of graphene oxide on cell migration, controversial reports exist in the literature. Bing et al. reported that graphene oxide slightly inhibited cytoskeletal reorganization in RAW264.7 cells.^[^ ^62^ ^]^ Wierzbicki et al. found that graphene oxide can disrupt cell adhesion and migration.^[^ ^63^ ^]^ Frontinan–Rubio et al. demonstrated that graphene oxide increases intracellular reactive oxygen species levels, leading to cytotoxicity and impaired cell migration in HaCaT cells.^[^ ^64^ ^]^ While our study demonstrated a slight increase in migration after 24 h of treatment with graphene oxide as compared to the control group, this effect was not observed at 48 h. Although Frontinan–Rubio et al. reported inhibitory effects on migration within the first 24 h, our study showed this effect on HFF cells after 48 h Considering the role of graphene oxide size, oxidation level, and surface chemistry, as well as the differences in cell types used (HFF cells in our study), further studies are needed to investigate the precise effects of graphene oxide based on its characteristics and its impact on various cell types. Quercetin, a potent antioxidant flavonoid, can enhance wound healing by promoting cell migration. The recent findings of Yi et al., who developed quercetin microneedles to promote wound healing, showed that the extract from these microneedles significantly increased migration in HUVEC and HaCaT cells after 24 h of treatment, aligning with our results. Moreover, they observed higher migration rates in HaCaT cells compared to HUVEC cells. Additionally, their study demonstrated that quercetin‐loaded microneedles significantly enhanced cell migration in vivo and promoted wound healing.^[^ ^65^ ^]^ A study conducted by Chittasupho and colleagues investigated the effects of quercetin, curcumin, and their combination on wound healing and fibroblast migration in vitro. The results indicated that quercetin alone exhibited a higher radical scavenging activity and wound closure compared to curcumin or their combination.^[^ ^66^ ^]^ Additionally, the findings of Irfan and coworkers showed that quercetin significantly increased cell migration in mesenchymal stem cells.^[^ ^67^ ^]^ Our study demonstrated that quercetin's efficacy in promoting cell migration was more pronounced 48 h post‐treatment. According to the cumulative release profile of quercetin from graphene oxide, as shown in Figure 2, 25.7 ± 1.37% of quercetin was released within 24 h. While this is a relatively small amount compared to free quercetin (10 μg mL^−1^), it was released slowly and exhibited better effects, although not statistically significant. However, after 48 h, with an increased amount of released quercetin, its effect on wound closure was significantly greater compared to free quercetin. A recent study by Liu et al. demonstrated that the use of chitosan‐coated zein/shellac nanoparticles as a nanocarrier for quercetin delivery enhanced the antioxidant capacity of quercetin.^[^ ^68^ ^]^ The use of nanocarriers can increase the solubility and bioavailability of quercetin, protect it from enzymatic degradation, and enable controlled release, ultimately enhancing its biological effects, including antioxidant and anti‐inflammatory properties, and promoting cell migration.^[^ ^69^ ^]^ Based on these findings, it can be concluded that graphene oxide, as an efficient nanocarrier, significantly enhanced the efficacy of quercetin in promoting cell migration and wound closure.

### 3.5 Determination of MIC and MBC

One complication that can arise particularly in chronic wounds is bacterial infection. Factors such as reduced blood supply, impaired immune function, and chronic inflammation can create an environment conducive to bacterial growth in wounds, delaying healing, and in severe cases, spreading to surrounding tissues and posing serious risks.^[^ ^70^ ^]^ In this study, to investigate the antimicrobial properties of graphene oxide and quercetin‐loaded graphene oxide against *S. aureus* and *E. coli*, the MIC and MBC were determined. The MIC and MBC values for the samples are shown in **Table** **3** and **4**. As the results indicate, the MIC of graphene oxide against *S. aureus* and *E. coli* was 9.7 and 19.5 μg mL^−1^, respectively, demonstrating that *S. aureus* was more sensitive to graphene oxide compared to *E. coli*. Furthermore, based on the MIC results, the quercetin‐loaded graphene oxide exhibited a MIC of 4.8 μg mL^−1^ against both bacteria, indicating an increase in the antibacterial properties of graphene oxide after loading with quercetin. The results showed that the MBC obtained for quercetin‐loaded graphene oxide against both bacteria was also 4.8 μg mL^−1^, which is (equal) to the MIC value (Figure S3, Supporting Information). Gao et al. found that graphene oxide can wrap bacteria, causing cell membrane damage and inhibiting bacterial growth. Their observations showed that this effect was stronger against *S. aureus* as compared to *E. coli*. Gram‐negative bacteria like *E. coli*, with their outer membrane, exhibit greater resistance to graphene oxide wrapping and damage compared to Gram‐positive bacteria like *S. aureus* lacking an outer membrane. Additionally, smaller bacteria are more susceptible to graphene oxide‐induced damage than larger bacteria. Increasing the concentration of graphene oxide leads to greater membrane damage in both bacteria, which is more lethal for *S. aureus*. These results align with our findings.^[^ ^71^, ^72^, ^73^ ^]^ Previous studies have reported varying MIC values for quercetin, ranging from 64–2476.4 μg mL^−1^ for *E. coli* and 20‐2053.6 μg mL^−1^ for *S. aureus.* ^[^ ^74^, ^75^, ^76^, ^77^ ^]^ In our study, loading quercetin onto graphene oxide enhanced the antibacterial properties of graphene oxide, which is consistent with previous research.^[^ ^71^, ^78^ ^]^ Quercetin exerts its antibacterial properties through mechanisms such as disruption of bacterial cell walls and cell membranes, disruption of nucleic acid synthesis, and inhibition of biofilm formation.^[^ ^79^ ^]^ Loading Quercetin onto graphene oxide can enhance these effects on bacteria. The increase in the antibacterial properties of graphene oxide through loading with another flavonoid, such as curcumin, has also been investigated. In a study by Bugli et al., loading curcumin onto graphene oxide with loading efficiencies of 29%, 60%, and 88% resulted in MIC values of 18.75, 4.7, and 2.35 μg/mL, respectively, against methicillin‐resistant *Staphylococcus aureus*. This study showed that while graphene oxide alone simply wraps around bacterial cells, curcumin‐loaded graphene oxide causes damage and disruption of the bacterial membrane.^[^ ^80^ ^]^ Overall, these results suggest that loading quercetin onto graphene oxide increases the antibacterial properties of graphene oxide against *S. aureus* and *E. coli*, making it a promising candidate as a material with potent antibacterial properties.

**Table 3:** MIC values of GO and quercetin‐loaded GO.

Strain/treatment	GO [μg/mL]	GO/Qu [μg/mL]
*S. areus*	9.7	4.8
*E. coli*	19.5	4.8

**Table 4:** MBC values of GO and quercetin‐loaded GO.

Strain/treatment	GO [μg/mL]	GO/Qu [μg/mL]
*S. areus*	19.5	4.8
*E. coli*	39.05	4.8

## 4 Conclusion

**Figure 6:**
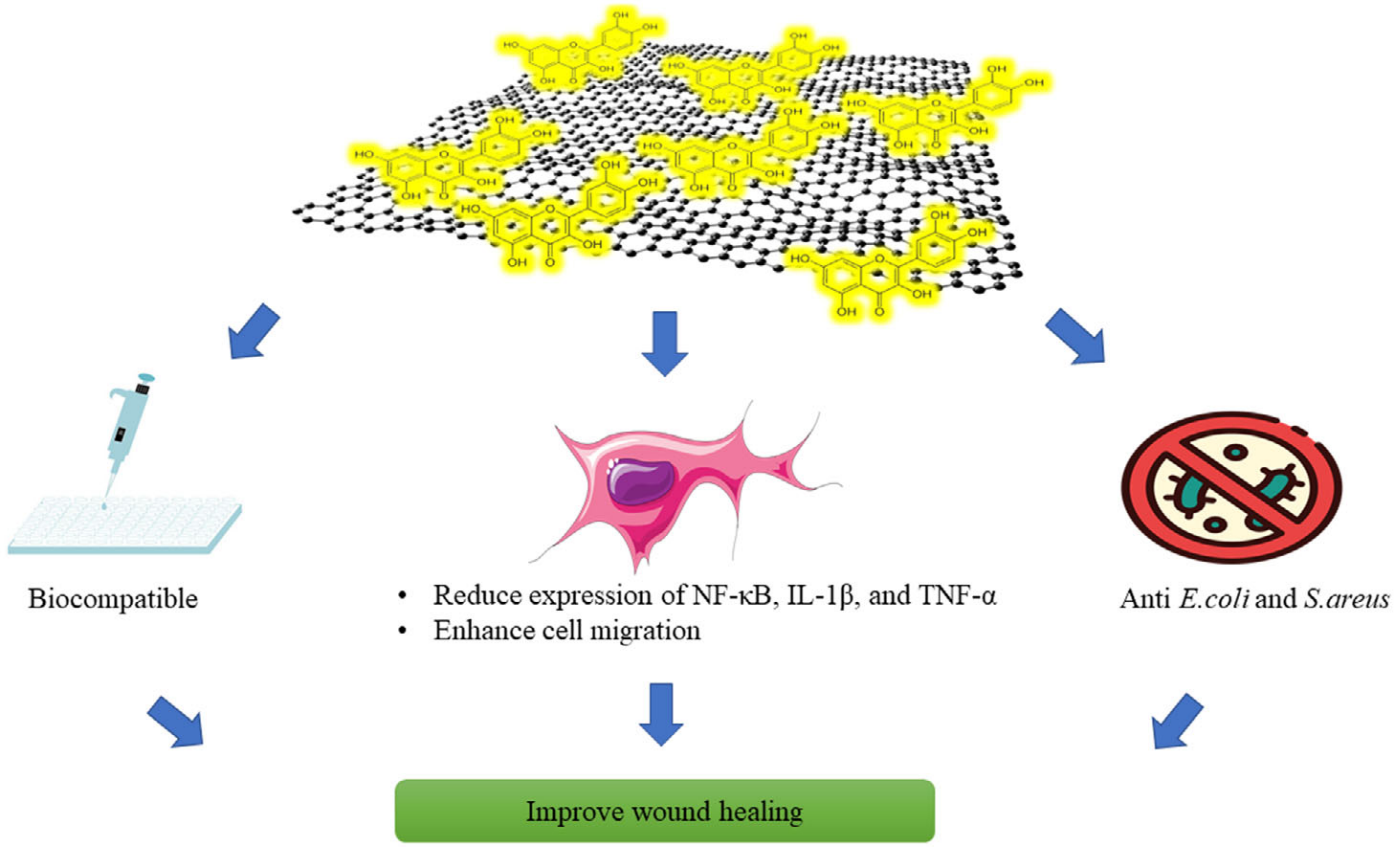
Biological effects of quercetin‐loaded GO for wound healing.

Our study aims to investigate the in vitro biological effects of quercetin‐loaded GO for wound healing. Our results demonstrated that GO can facilitate the sustained release of quercetin, and this release is more pronounced at an alkaline pH, which is indicative of infected wounds, compared to pH 7.4. Additionally, quercetin‐loaded GO exhibited excellent biocompatibility and, at a concentration of 10 μg mL^−1^ used in this study, significantly downregulated the expression of three genes, NF‐κB, IL‐1*β*, and TNF‐*α*, which play a crucial role in regulating inflammation. Furthermore, it significantly accelerated wound closure in a scratch assay. Moreover, quercetin‐loaded GO, with a MIC and MBC of 4.8 μg/mL, can (lead) to infection inhibition in the wound area. **Figure** **6** demonstrates the biological effects of quercetin‐loaded GO. Future studies are recommended to investigate the mechanism of the biological effects of quercetin‐loaded GO and its influence on various cell types, including macrophages. Additionally, its potential for wound dressing applications should be explored in vivo.

## Conflict of Interest

The authors declare no conflict of interest.

## Supporting information

Supplementary Material

## Data Availability

The data that support the findings of this study are available from the corresponding author upon reasonable request.
