# Supplementary material for: Quercetin‐Loaded Graphene Oxide Nanoparticles Modulate Inflammatory Gene Expression and Enhance Cell Migration In Vitro
Source: ChemistryOpen. 2025 Jul 23;14(11):e202500215. doi: 10.1002/open.202500215 (PMC12598803; doi:10.1002/open.202500215)
Supplement: Supplementary file 1 — Supplementary Material [file OPEN-14-e202500215-s001.pdf]

Supporting information

a

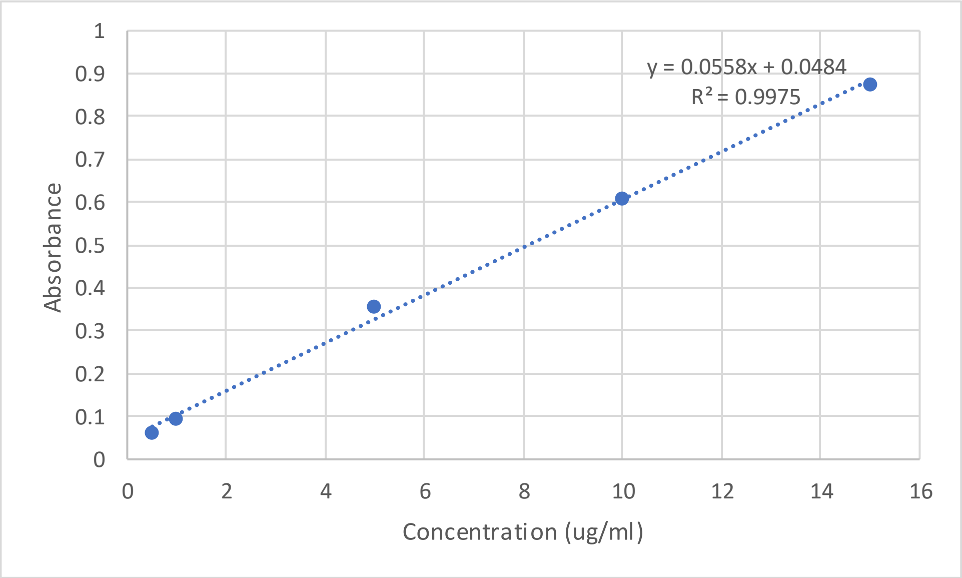

b

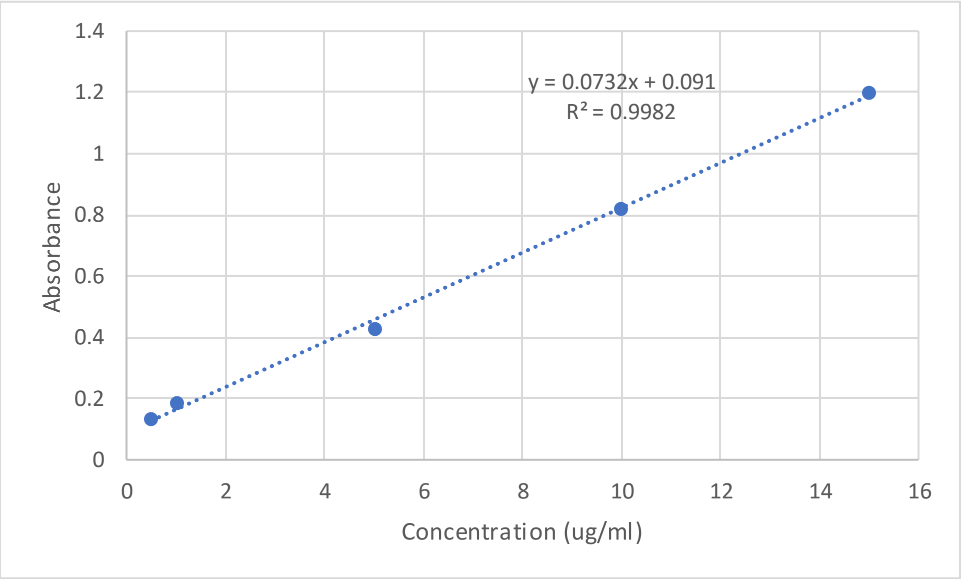

Figure S1. Calibration curves of quercetin at (a)pH 7.4 and (b) pH 8.5.

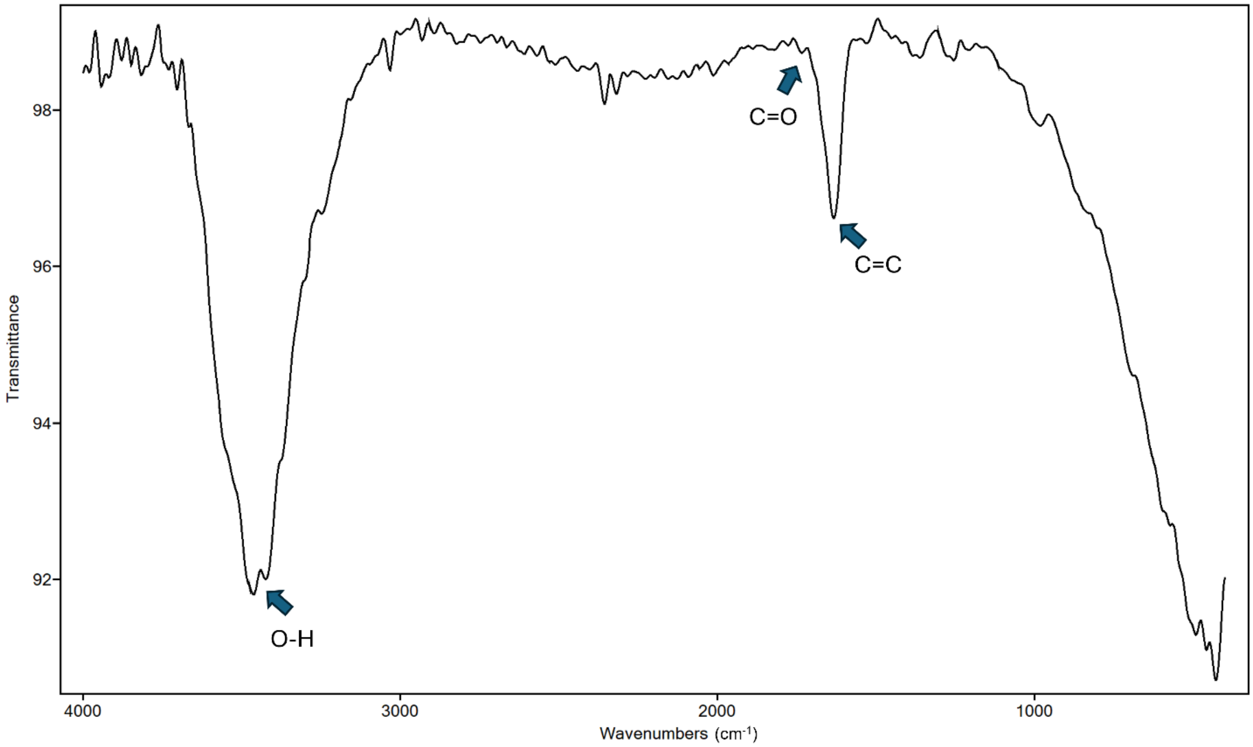

Figure S2. FTIR spectra of GO

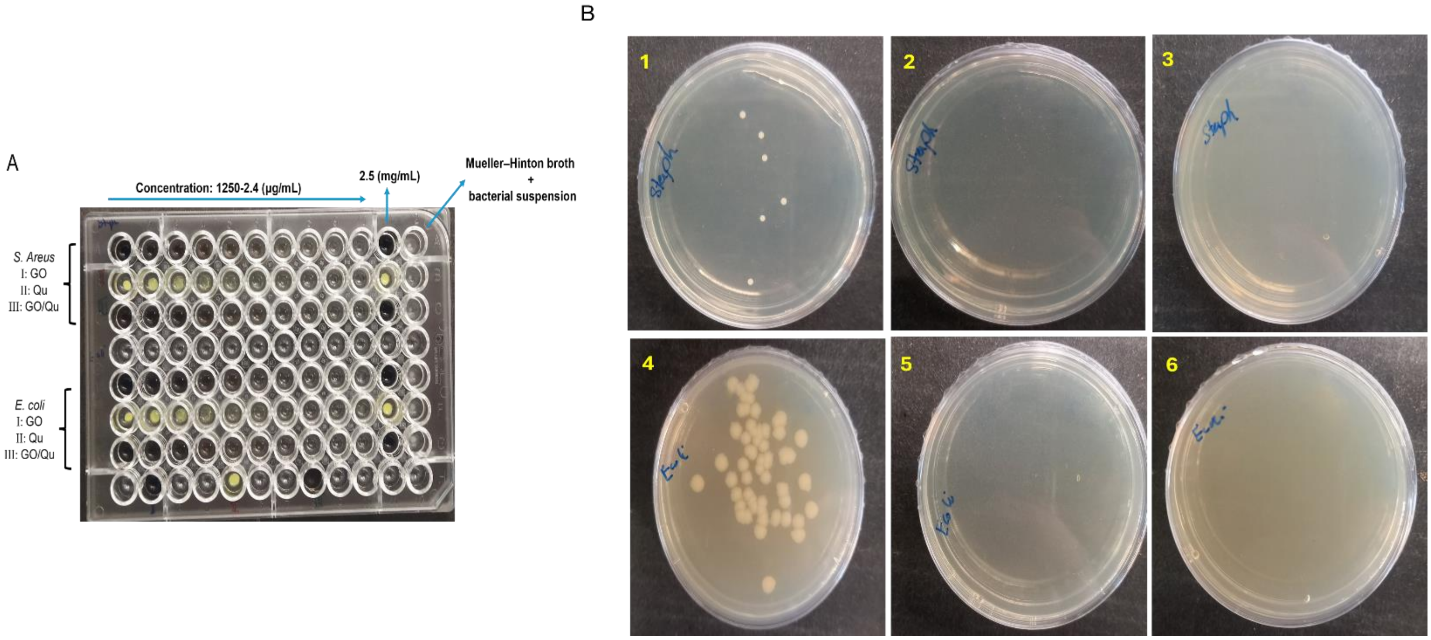

Figure S3. **A)** Representative image of a 96-well microplate used for the MIC (minimum inhibitory concentration) assay. The tested groups were incubated with *S. aureus* and *E. coli* bacterial suspensions in Mueller–Hinton broth at 37 ℃ for 24 hours. **B)** Representative agar plates used for MBC (Minimum Bactericidal Concentration) determination following MIC assay. 50 ul aliquot from specific wells of the 96-well microdilution plates were seeded on Mueller–Hinton agar plate and incubated for 24 h at 37 ℃. Plates 1–3 show *S. aureus* results: (1) GO sample (from MIC well), (2) GO sample (pre-MIC concentration, 19.5 µg/mL), (3) GO/Qu sample (from MIC well). Plates 4–6 show *E. coli* results: (4) GO sample (from MIC well), (5) GO sample (pre-MIC concentration, 39.05 µg/mL), (6) GO/Qu sample (from MIC well).
